# Recurrent Urolithiasis Revealing Primary Hyperparathyroidism in a Nephrology Department

**DOI:** 10.1155/2024/1265364

**Published:** 2024-02-21

**Authors:** Hajji Meriam, Hayet Kaaroud, Rahma Karray, Fethi Ben Hamida, Kahena Bouzid, Ezzeddine Abderrahim

**Affiliations:** ^1^Department of Medicine A, Charles Nicolle Hospital, Tunis, Tunisia; ^2^Kidney Pathology Laboratory LR00SP01, Charles Nicolle Hospital, Tunis, Tunisia; ^3^Faculty of Medicine of Tunis, El Manar University, Tunis, Tunisia; ^4^Department of Biochemistry, Charles Nicolle Hospital, Tunis, Tunisia

## Abstract

**Background:**

Urinary lithiasis constitutes a recurrent pathology affecting a relatively young population. The risk of progression to chronic renal failure and the cost of treatment are the most important issues. Primary hyperparathyroidism (PHPT) is responsible for urolithiasis and nephrocalcinosis in 7% of patients, and it represents the 7th cause of urolithiasis in Tunisia. Unfortunately, it remains an underdiagnosed pathology although it is curable. We aim to determine the clinical, biological, therapeutic, and evolutionary particularities of urinary lithiasis associated with PHPT in a nephrology setting.

**Methods:**

This is a monocentric, retrospective, descriptive study which took place in our nephrology department during the period from January 2010 to January 2023. Ten patients were included. All of them underwent blood and urine tests and a morphoconstitutional study of the urinary stones if possible.

**Results:**

The median age at diagnosis of PHPT was 42 years (34–54). The median time from the onset of kidney stones to the diagnosis of PHPT was 6.2 years (1–17). The male/female gender ratio was 0.66. Five patients had hypertension, two patients had obesity, one patient had diabetes, and three patients had urinary tract infections. Kidney stones were bilateral in eight cases and unilateral in two cases. Nine patients underwent urological intervention: surgery in 5 cases associated with nephrectomy in one case, extracorporeal lithotripsy in 4 cases, and percutaneous nephrolithotomy in two cases. The diagnosis of PHPT was retained with high or uncontrolled PTH associated with hypercalcemia in 8 cases and normocalcemic PHPT was found in 2 patients. Two patients had parathyroid adenoma and one patient had mediastinal adenoma. Radiology exploration was normal for the others patients. Surgery was performed in 7 patients and histology revealed an adenoma in 5 cases and hyperplasia in one case. The predominant urinary risk factors in our study were hypercalciuria in 6 cases and insufficient diuresis in 4 cases.

**Conclusion:**

This study underlines the role of the nephrologist in the exploration of urinary lithiasis and the prevention of recurrences, especially as PHPT is a curable aetiology of urolithiasis and affects a relatively young population. The determination of the epidemiological profile of patients with stones associated with primary PHPT and lithogenic risk factors allows the primary and secondary prevention of stone formation.

## 1. Introduction

Recurrent urolithiasis requires exploration to determine aetiology and institute therapy. Unfortunately, primary hyperparathyroidism (PHPT) remains an underdiagnosed pathology although it is curable. In Tunisia, few studies have dealt with this subject. We aim to determine the clinical, biological, therapeutic, and outcome particularities of recurrent urolithiasis, revealing PHPT.

## 2. Materials and Methods

### 2.1. Study Design

This is a monocentric, retrospective, descriptive study which took place in our nephrology department during the period from January 2010 to January 2023. The patients with recurrent urolithiasis were referred for an etiological assessment. For each patient, his medical and surgical file history was recorded, including his medical history (diabetes mellitus and gout), history of urolithiasis, including their treatments and social history (smoking and alcohol consumption). Metabolic stone workup included a 24-hour urine collection measure of volume, pH, and creatinine excretion over 24 hours, calcium, sodium, phosphate, uric acid, citrate, oxalate, magnesium, and urea nitrogen. In addition, serum dosages of bicarbonate, glucose, urea, creatinine, magnesium, phosphate, normalized calcium, uric acid, parathyroid hormone (PTH), and 25-(OH) D were obtained. The calcium/creatinine clearance ratio (CCCR) was calculated as (24-hour urine calcium/total plasma calcium)/(24-hour urine creatinine/plasma creatinine). GFR was estimated using the MDRD formula (Modification of the Diet in Renal Disease). When the urinary stone was available, a morphological examination with an analysis by Fourier transform infrared spectroscopy (FTIR) was carried out. In the case of renal tubular acidosis, we retained HPTP after a negative etiological. Regarding the radiological exploration of the parathyroid gland, ultrasound was performed in 6 cases, sestamibi scan was performed in 7 cases, magnetic resonance imaging was performed in 2 cases, and CT scan was performed in 1 case. Bilateral neck exploration with direct visualization of the 4 parathyroid glands before the surgical removal of the hyperfunctioning parathyroid tissue was performed systematically. Inclusion criteria were patients in whom the diagnosis of PHPT has been retained. We excluded patients with secondary or tertiary PHPT. On a pre-established sheet, we collected the epidemiological, clinical, blood, and urine tests, urinary stones analysis, radiological data, the treatment, and the outcome.

### 2.2. Definitions

The diagnosis of PHPT was based on the elevated calcium and elevated or inappropriately normal PTH level [1].

Normocalcemic primary hyperparathyroidism (NPHPT) was defined as consistently elevated serum parathyroid hormone (PTH) levels with normal serum calcium concentration, after excluding secondary causes of PTH elevation such as vitamin D deficiency, kidney disease, malabsorption, and idiopathic hypercalciuria.

Vitamin D (VD) deficiency and VD insufficiency were defined as 25-(OH) VD levels ≤20 ng/mL and 21–29 ng/mL, respectively.

Chronic renal failure (CRF) was defined according to the KDIGO 2012 recommendations by the decrease in glomerular filtration rate (GFR) < 60 ml/min/1.73 m^2^ more than 3 months [2].

Hypertension (HT) is defined by a systolic blood pressure ≥140 mmHg and/or a diastolic pressure ≥90 mmHg. According to the 2018 recommendations of the European Society of Cardiology [3], Trachyspermum ammi (L.) Sprague (TAS) is an annual aromatic and herbaceous plant of the family *Apiaceae* [4].

## 3. Results

We enrolled 10 patients with a mean age at diagnosis of PHPT 42 years (34–54). The mean time from the onset of kidney stones to the diagnosis of PHPT was 6.2 years (1–17). They are six women and four men with a male/female gender ratio of 0.66. With respect to co-occurring medical conditions, five patients had hypertension, two patients had obesity, one patient had diabetes, and three patients had urinary tract infections (UTI). Kidney stones were bilateral in eight cases and unilateral in two cases. Nine patients underwent urological intervention: surgery in 5 cases was associated with nephrectomy in one case, extracorporeal lithotripsy in 4 cases, and percutaneous nephrolithotomy in two cases (Table 1). The laboratory parameters of our patients are summarised in Table 2. The mean serum PTH was 266.28 pg/mL (range: 126.9–605 pg/mL, ref range: 26.5–96.5 pg/mL). The mean serum 25(OH) D level was 17.3 ng/ml (range: 9.4–52.4 ng/ml). Five patients had VD deficiency and three patients had VD insufficiency.

Hypercalcemia was found in 7 patients with mean serum total calcium of 2.73 (range 2.4–3, reference range: 2.25–2.60 mmol/l). Hypophosphatemia was found in 8 patients (range 0.38–0.74 mmol/l, reference range: 0.74–1.52 mmol/l). Hyperuricemia was found in 5 patients (range 205–514 *µ*mol/l, reference range: 200–420 *µ*mol/l).

The most prevalent abnormalities in the 24-hour urine collection, in decreasing frequency, were hypercalciuria in 6 cases (range 0.8–13.28 mmol/24 h, reference range: 2.5–7.5 mmol/24 h). Suboptimal urine volume <2 L/day in 4 cases, hyperoxaluria in 3 cases (range 0.119–0.734 mmol/24 h, reference range: 0.1–0.5 mmol/24 h), hypocitraturia in the absence of an intercurrent urinary infection (<1.5 mmol/24 h) in 2 cases (0.11–4.5 mmol/24 h), and hyperuricosuria in 2 cases (range 1.35–4.9 mmol/24 h, reference range: 1.5–4.2 mmol/24 h). Four patients had positive crystalluria. Excessive salt intake was noted in five cases and excessive protein intake was noted in one case.

The diagnosis of PHPT was retained with high or uncontrolled PTH associated with hypercalcemia in 8 cases, and normocalcemic PHPT was found in 2 patients.

Two patients had parathyroid adenoma and one patient had mediastinal adenoma. Radiology exploration was normal for the others patients (Table 3). Surgery was performed in 7 patients, and histology revealed an adenoma in 5 cases, hyperplasia in one case, and the last patient was operated on in another hospital, and we do not disclose the details (Table 4).

## 4. Discussion

PHPT is the third most common endocrine disorder after diabetes mellitus and hypothyroidism [5]. Recent estimates suggest that 7–18% of patients with PHPT have kidney stones and 2–8% of patients with kidney stones have PHPT [6]. The mean age of PHPT diagnosis varies from 33 to 60 years which agrees with our data. Male PHPT patients are more likely than women to present with kidney stones, while the most common symptom of PHPT among female patients is osteoporosis [7]. According to our study, females exhibited a predominant predisposition to kidney stones formation. PHPT is caused by a solitary parathyroid adenoma in 80% of cases, whereas four-gland hyperplasia accounts for 10–15% [4].

Our results are consistent with data from the literature with a predominance of parathyroid adenoma. As described previously, we noted metabolic complications during hyperparathyroidism in patients such as arterial hypertension, diabetes, and obesity [5, 8, 9]. UTI occurred in 20% of patients with urolithiasis. Our results agree with that, since 3 of our patients had a history of recurrent UTI. The main renal manifestations of PHPT are hypercalciuria and nephrolithiasis. PHPT must always be evaluated in patients with clinical histories of recurrent nephrolithiasis or nephrocalcinosis.

All our patients had recurrent urolithiasis but they were referred late with a delay of 6.2 years. The results of our study demonstrate the need for better knowledge of PHPT and better adherence to guidelines for metabolic assessment of kidney stones.

Diagnosis of PHPT is mainly biochemical with hypercalcemia and increased or uncontrolled PTH. However, normocalcemic PHPT has prevalence between 0.5% and 16% [10], and it was identified in 2 of our patients. The pathophysiology of NPHPT is unknown. The most widely accepted concept is that NPHPT is an early form of PHPT, and hypercalcemia can appear in 19% of cases within 3 years [11]. The prevalence of VD inadequacy is higher among PHPT patients than in the general population [12, 13]. Regarding our patients, 5 had VD deficiency and 3 had VD insufficiency. One patient had a normal level, and the other had no test available. To prevent postoperative hypocalcemia and hungry bone syndrome, supplementation vitamin D is important with doses of cholecalciferol ranging from 600 to 1000 IU per day, targeting vitamin D levels of 50–75 nmol/L.

Stone-forming patients are strong calcium excreters [14]. This finding is in concordance with the pathophysiology of stone formation in PHPT since hypercalciuria was identified in 9 of our patients. However, some PHPT hypercalciuric patients do not develop kidney stones. Other urinary and nonurinary risk factors not yet clearly defined seem to contribute to the development of lithiasis disease. The predominant urinary risk factor in our study was hypercalciuria in 6 cases and insufficient diuresis in 4 cases (Table 2).

The prevalence of renal dysfunction (estimated glomerular filtration rate (eGFR) < 60 ml/min) is low, around 15 to 17%. Neither history of nephrolithiasis nor PHPT severity was a risk factor for reduced eGFR [15]. Traditional risk factors, such as age, diabetes, and hypertension, were associated with lower kidney function. One obese woman in our series had a chronic renal disease.

During PHPT, the mainly component of urinary stones is either calcium or phosphorus and the association of the 2 components is noted in 58.3% of cases and significantly correlated to PHPT (*p* < 0.0001) [16]. Among the 7 renal stones analyzed, the majority component was calcium (50–70%) in 5 cases and phosphate (70%) in 2 cases. Distal tubular acidosis, noted in two of our patients, is a complication of PHPT and may contribute to phosphatic renal lithiasis.

In our study, the oxalic component was more frequent than that reported in the literature. This could be explained by the oxalate-rich Mediterranean diet and/or the use of parsley or celery infusions, as in 3 cases in our series. According to a Tunisian epidemiological study, 79% of patients with oxalocalcic stones used to take parsley and/or celery-based infusions [17]. Parathyroid imaging assists the parathyroid surgeon in identifying the anatomic position of abnormal glands [18]. Parathyroid ultrasound (US) and parathyroid scintigraphy (PS) are the main methods used for this purpose. The complementary role of both these tests is emphasized in the literature. A positive imaging was noted in 4 of 7 patients explored. Parathyroidectomy should be recommended in all patients with kidney stones to reduce their risk of recurrence [19]. Six of our patients underwent surgery. With the surgical cure of PHPT, serum biochemistries normalize and urinary calcium levels decline.

Among the 5 patients followed for a mean time of 4 years, blood and urine calcium levels decreased in all cases. All patients remained persistently hyperparathyroid after successful parathyroid surgery as evidenced by normalization of serum and urine calcium levels due to untreated vitamin D deficiency or inadequate calcium intake [20].

The strengths of our study lie in the establishment of a specialized consultation within our department for lithiasis management since 2008. This facilitated the department's ability to conduct an etiological diagnosis in collaboration with the biochemistry laboratory, providing valuable insights into lithiasis recurrence over several years. However, the study does have certain limitations, including a small sample size, inadequate assessment of lithiasis, and occasional patient neglect of follow-up and consultation appointments.

## 5. Conclusion

In the case of recurrent lithiasis, the phosphocalcic assessment should be carried out as a first-line investigation, according to the guidelines. Any metabolic abnormality that suggests primary hyperparathyroidism should prompt referral to a specialist, especially since it is a potential cause of recurrence. Our work highlights the importance of timely referral to a specialist.

## Figures and Tables

**Table 1:** Clinical features of our study population.

Patients	Age (years)/gender	Medical history	Herbal infusion	BMI/Weight (kg)	Delay of diagnosis (years)	Kidney stone's location	Spontaneous emission of stones	Urological intervention	Kidney stone analysis
1 (WR)	39/M	HP-UI	Yes	29.93/92	8	Bilateral		ECL-PCNL	Ox-Ca
									Ox: 73%, Ca 20%

2 (BA)	48/M	HP	—	26.2/77	17	Unilateral		ECL-surgery	Ox-Ca
									Ca: 60%, Ox: 22%
									Infectious component

3 (DH)	47/F	HP	—	26.47/76	6	Bilateral	Yes	No	Ox-Ph-Ca
									Ca: 70%, Ph: 20%, Ox: 8%

4 (HR)	38/F	No	—		2	Bilateral	Yes		Ox-Ph-Ca
									Ca: 75%, Ph: 20%, Ox: ..%

5 (NL)	48/F	HP diabetes	—	32.23/75	1	Bilateral		ECL-PCNL surgery	Ph-Ca
									Ca: 50%, Ph: 38%

6 (GK)	45/F	HP-UI	Yes	29.26/71	5	Bilateral		ECL-surgery	Ph: 70%
									Infectious component

7 (BAS)	27/H	No		-/93	5	Bilateral		ECL	ND

8 (NH)	44/H	No		-/87	2	Unilateral	Yes	No	Ca-Ph
									Ca: 85%, Ph: 15%

9 (DS)	54/F	No	Yes	-/75	—	Bilateral		Surgery	Ph-Ox-Ca
									Ph: 70%, Ox: 17%,
									Ca: 10%
									Infectious component

10 (HI)	34/F	UI *E. coli*	No	52/141	10	Bilateral	Yes	Surgery neprectomy	ND

ECL: extra corporeal lithotripsy, PCNL: percutaneous nephrolithotomy, and ND: none done.

**Table 2:** Laboratory parameters of our study population.

	Biological blood data									Urine testing data										
	Ca (mmol/l)	Ph (mmol/l)	HCO_3_^−^ (mmol/l)	Creatinine (*µ*mol/l)	GFR (ml/mn/1.73 m^2^ sc)	Uric acid (*µ*mol/l)	PTH (pg/ml)	Protidemia/albuminemia (g/l)	25 OH Vit D3 (ng/ml)	Ca (mmol/24 h)	Ph (mmol/24 h)	Uric acid (mmol/24 h)	Ci (mmol/24 h)	OX (mmol/24 h)	pH value	Diuresis (l)	Salt intake (g/day)	Protein intake (g/kg/day)	UCBE	Crystalluria
1 (WR)	2.4	0.74	23	66	124	266	140	-/42	<7	10.26	24	4.9	4.5	0.540	5.9	2.7	15	0.7	Neg	Wd
2 (BA)	2.45	0.69	26	72	107.5	379	160	69/-	24.4	9.2	32.8	2.46	1.99	0.3	5.6	2.4	8.3	0.8	Neg	Wd
3 (DH)	3	0.38	—	60	80.14	334	605	78/40	33.58	4.2	21.2	2.55	—	—	—	1.3	—	—	Neg	ND
4 (HR)	2.89	0.58	23	66	92.51	255	409	80/-	<7	5.1	12	1.35	0.73	0.119	5.8	1	5	0.4	Nege	Negative
5 (NL)	2.8	0.73	24	65	89.79	327	215		19.8	2.72	25.1	2.17	0.11	0.3	6	1.6	6	0.6	Neg	ND
6 (GK)	2.65	0.69	21	80	71.59	494	212	-/43	21.3	7.9	21.3	3.52	0.48	0.716	9	2.5	9.6	1.1	Pos	Wd + Struvite
7 (BAS)	2.78	0.65	—	77	111.8	452	126.9		20.32	9.68	42.8	4.9	—	—	—	3.4	6.2	—		ND
8 (NH)	2.68	0.52	—	79	98.35	379	245.9		15.9	13.28	44	—	—	—	—	2			Neg	ND
9 (DS)	2.75	0.69	22	87	84.4	205	229	80/-	ND	7.5	15	2.74	1.45	0.230	6.9	5	5.9	0.6	Neg	ND
10 (HI)	2.92	0.69	14	149	36.9	514	320	78/-	14.9	0.8	30.4	2.22	0.59	0.734	7.2	1.6	8.4	0.6	Pos	Struvite

Ca: calcium, Ph: phosphate, GFR: glomerular filtration rate, Ci: citrate, Ox: oxalate, Wd: Wedellite, ND: none done, Neg: negative, Pos: positive, UCBE: urine cytobacteriological examination, and l: litre.

**Table 3:** Radiological exploration of parathyroids.

Patients	Ultrasound	Scintigraphy	MRI	TDM
1 (WR)	Normal	Normal	—	—
2 (BA)	Normal	Right upper mediastinal ectopic adenoma	Right upper mediastinal ectopic adenoma measuring 5 mm in diameter	—
3 (DH)	Normal	Normal	Lower left parathyroid adenoma measuring 16 mm in diameter	—
4 (HR)		Lower left parathyroid adenoma	Lower left parathyroid adenoma	—
5 (NL)	Normal	Normal	Normal	—
6 (GK)	Normal	Normal	—	Normal
7 (BAS)	ND	ND	ND	—
8 (NH)	ND	ND	ND	—
9 (DS)	ND	ND	ND	—
10 (HI)	Left parathyroid hyperplasia	Adenoma	—	—

MRI: magnetic resonance imaging, TDM: tomodensitometry, and ND: none done.

**Table 4:** Prognostic data of our study population.

Patients	Medical treatment	Surgical treatment	Histology	Follow-up
1 (WR)	25 OH Vit D3	No	—	**6 Years**
				PTH: 114 pg/ml
				Ca: 2.36 mmol/l
				Ph: 0.88 mmol/l
				25 OH Vit D: 28.7 ng/ml
				CaU: 8.3 mmol/24 h
				PhU: 21 mmol/24 h

2 (BA)	25 OH Vit D3	Yes Removal of the subpolar lower parathyroid	Parathyroid hyperplasia	**3 Years**
				CT scan of the parathyroids 5 months after surgery: 16 mm mediastinal adenoma not reoperated
				PTH: 96.3 pg/ml
				Ca: 2.7 mmol/l
				Ph: 0.71 mmol/l
				CaU: 3 mmol/24 h
				PhU: 13.5 mmol/24 h

3 (DH)	25 OH Vit D3	Yes Removal of a 2 cm nodule from the left lower parathyroid	Parathyroid adenoma	**1 year**
				PTH: 96.3 pg/ml
				25 OH Vit D3: 39 ng/ml Ca: 2.27 mmol/l
				Ph: 0.73 mmol/l
				CaU: 3 mmol/24 h
				PhU: 13.8 mmol/24 h

4 (HR)		Yes	Parathyroid adenoma	PLTF

5 (NL)		Yes Parathyroidectomy of the lower and upper left, upper right, and half of the lower right parathyroid	Parathyroid adenoma	**8 years**
				PTH: 30 pg/ml
				Ca: 2.26 mmol/l
				Ph: 1.39 mmol/l
				Ca u: 1.4
				Ph u: 14.8

6 GK		Yes Right lower parathyroid adenoma	Parathyroid adenoma	**3 years**
				Creat: 108
				Ca: 2.33 mmol/l
				Ph: 0.98 mmol/l
				PTH: 74.05 pg/ml
				Ca U: 2.08 mmol/24 h
				Ph U: 16 mmol/24 h

7 (BAS)		Yes Parathyroidectomy	ND	PLTF

8 (NH)		—	ND	PLTF

9 (DS)		—	ND	PLTF

10 (HI)		Yes Removal of a 2.5 cm parathyroid adenoma	Parathyroid adenoma	**3.5 Years**
				Creat: 160 *µ*mol/l
				PTH: 300 pg/ml
				Ca: 2.18 mmol/l
				Ph: 1.03 mmol/l
				25 OH Vit D3: 16 ng/ml

PLTF: patient lost to follow-up: ND: none done, PTH: parathormone, Ca: calcium, Ph: phosphate, U: urinary, and Creat: serum creatinine. Bold values represent the follow-up period for each patient.

## Data Availability

The data used to support the findings of this study are included within the article.
